# Mechanisms protect airborne green microalgae during long distance dispersal

**DOI:** 10.1038/s41598-020-71004-y

**Published:** 2020-08-19

**Authors:** Chia-Sheng Chiu, Pai-Ho Chiu, Tze Ching Yong, Hsin-Pei Tsai, Keryea Soong, Hsiang-En Huang, Ching-Nen Nathan Chen

**Affiliations:** 1 Department of Oceanography, National Sun Yat-Sen University, Kaohsiung, 804 Taiwan; 2 Department of Life Science, National Taitung University, Taitung, 950 Taiwan

**Keywords:** Ecology, Ecophysiology

## Abstract

Viable microalgae occur in the air. Whether they can survive the stresses such as UV, desiccation and freezing temperatures at high altitudes during long distance dispersal is rarely studied. If yes, what mechanisms confer the tolerance? Four freshwater airborne green microalgae were isolated from Dongsha Atoll in the South China Sea, classified as *Scenedesmus* sp. DSA1, *Coelastrella* sp. DSA2, *Coelastrella* sp. DSA3 and *Desmodesmus* sp. DSA6 based on their morphologies and ITS sequences. Their survival rates under UV stress were tightly correlated with their cell wall thickness. All the four airborne green microalgae survived the air-dry stress on benchtop followed by − 20 °C freeze–desiccation stress for 4 weeks, but not the two waterborne green microalgae *Desmodesmus* sp. F5 and *Neodesmus* sp. UTEX 2219-4 used as controls. Three of the four airborne microalgae survived the lyophilization treatment, excluding *Desmodesmus* sp. DSA6 and the two waterborne microalgae. The four airborne microalgae produced carotenoids under prolonged stress conditions, which might help detoxify the reactive oxygen species generated under environmental stresses and shield from the high-light stress in the air. Characterization of these airborne microalgae may help answer how the descendants of green algae survived on the land about 450 MYA.

## Introduction

Darwin described the falling of fine dust particles from the sails of his vessel on many occasions while cruising in the Atlantic Ocean. He reported that the particles contained dry “infusoria” which included siliceous shells of many freshwater species and a least one marine diatom identified using microscopes available at that time^1^. Although he did not attempt to grow microalgae from the dust collections, it is now well known that viable microalgae occur in the atmosphere^2–5^. In a historical survey of airborne microalgae, van Overeem collected green microalgae in northern Europe using an airplane at various altitudes^3^. These cells were cultivated, and nine algal isolates were identified. In the US, airborne microalgal cells were isolated in many states using car and airplane in a study conducted by Brown et al*.*^2^. These isolates were classified into 38 and 17 genera of Chlorophyta and Chrysophyta, respectively, in addition to seven genera of cyanobacteria. More recently, airborne algae were surveyed in Hawaii, and the results of this investigation showed that these algae were dominated by cyanobacteria, followed by green algae and diatoms^6^. A comprehensive review of the history of airborne microalgae studies is provided by Sharma et al.^4^.

Most studies on airborne microalgae have been focused on their diversity and abundance in various regions and at different altitudes, as well as the effects of these airborne cells on human health, such allergenicity and toxicity^4,7^. How they resist environmental stresses in the air has rarely been discussed^8^. When microalgal cells are dispersed through a long distance in the atmosphere, they encounter a few environmental stresses not common in water and on the moist surfaces of tree barks and rocks. These stresses include high levels and long durations of UV exposure, desiccation and freezing temperatures especially at high altitudes in wintertime. These stresses are lethal to most eukaryotic cells after prolonged exposure. Only a few types of eukaryotic cells such as fungal spores, pollen grains and zooplankton cysts^9^ besides microorganisms can survive long distance aerial dispersal. How microalgal cells resist these extreme stresses is still poorly understood. This knowledge might be useful for crop breeding since land plants are descendants of ancient green algae, thus crops and green algae share many common cellular mechanisms.

The location of Dongsha Atoll (20° 43′ N, 116° 42′ E), remotely isolated in the South China Sea, is ideal for collecting long distance dispersed airborne microalgae. The strong, seasonal and dry northeastern wind arrives at this atoll during the winter, and may bring particles from northeastern China, Korea, Japan, or Taiwan (see the wind map in Supplementary Figure 1). Wind directions in other seasons are more variable due to the changing locations of the atmospheric high-pressure systems. Therefore, there are plentiful chances for microalgae to arrive at this atoll carried by wind from afar after a long journey. A tiny island located at the west part of this atoll, Dongsha Island, does not have any freshwater ponds or rivers since its formation. Precipitation seeps into the coral sands or runs into the sea rapidly, therefore there is no ecosystem that can support evolution of freshwater microalgae on this island. Any freshwater microalgae isolated on this tiny island would be evolved from other lands with freshwater ecosystems. On the other hand, non-airborne dispersers could not survive the journey if there is no exogenous protection available to them to shield against the stresses (see in “Discussion” section). Whatever brought by the wind and could still survive on this tiny island are good candidates of true airborne dispersers in nature; this is especially true for those that could only grow in freshwater. The stresses encountered by these airborne microalgae would be similar to those faced by the primitive terrestrial plants when they first left the aquatic environments. Characterization of these airborne microalgae may help answer the evolutionary questions such as how the descendants of green algae survived on the non-shaded dry land and why algae in other phyla did not. Here, we report the isolation, characterization of the airborne freshwater microalgae from Dongsha Island and their stress tolerance to UV, desiccation and freezing temperatures, in an attempt to discover the potential mechanisms that confer the stress tolerance to airborne green microalgae.

## Results

### Isolation and identification of four airborne microalgal strains belonging to three genera from Dongsha Island in the South China Sea

**Figure 1:**
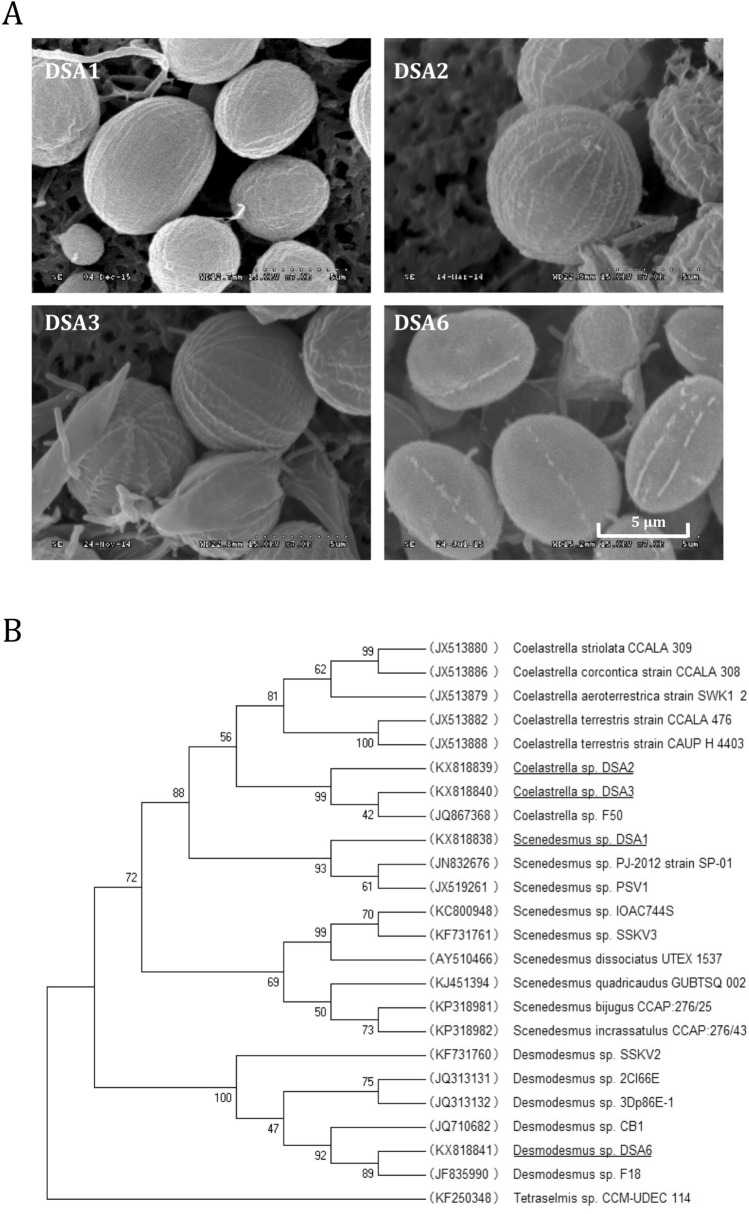
**A** Scanning electron microscopy (SEM) micrographs of the four airborne microalgae in the early stationary phase. Upper panel, left and right: *Scenedesmus* sp. DSA1 and *Coelastrella* sp. DSA2. Lower panel, left and right: *Coelastrella* sp. DSA3 and *Desmodesmus* sp. DSA6. **B** Phylogenetic analysis of the four airborne microalgal strains (underlined) and their related species based on the fusion sequences of ITS1 and ITS2 of each species. The numbers in the parentheses are accession numbers of each sequence in GenBank. The numbers at the nodes indicate bootstrap values (expressed as percentage) with 500 replicates.

Four microalgal strains belonging to three genera were successfully isolated in the collection trip in December 2013 and all the four could not grow in 2f seawater medium^10^ (in both 33 and 22‰ salt levels), indicating they were freshwater species. As shown in Fig. 1, based on the morphology revealed by Scanning Electron Microscopy and the phylogenetic analysis, the four strains were named *Scenedesmus* sp. DSA1 (stands for Dongsha airborne #1), *Coelastrella* sp. DSA2, *Coelastrella* sp. DSA3, and *Desmodesmus* sp. DSA6 (hereafter referred to as DSA1, DSA2, DSA3 and DSA6, respectively). All four strains are members of the family Scenedesmaceae in Chlorophyta. Sequences of the 18S rDNA and ITS1-5.8S-ITS2 fragments of the four airborne microalgal strains were deposited into GenBank under the accession numbers KX818834–KX818841.

### The airborne green microalgae had better UV tolerance than the waterborne green microalgae

**Figure 2:**
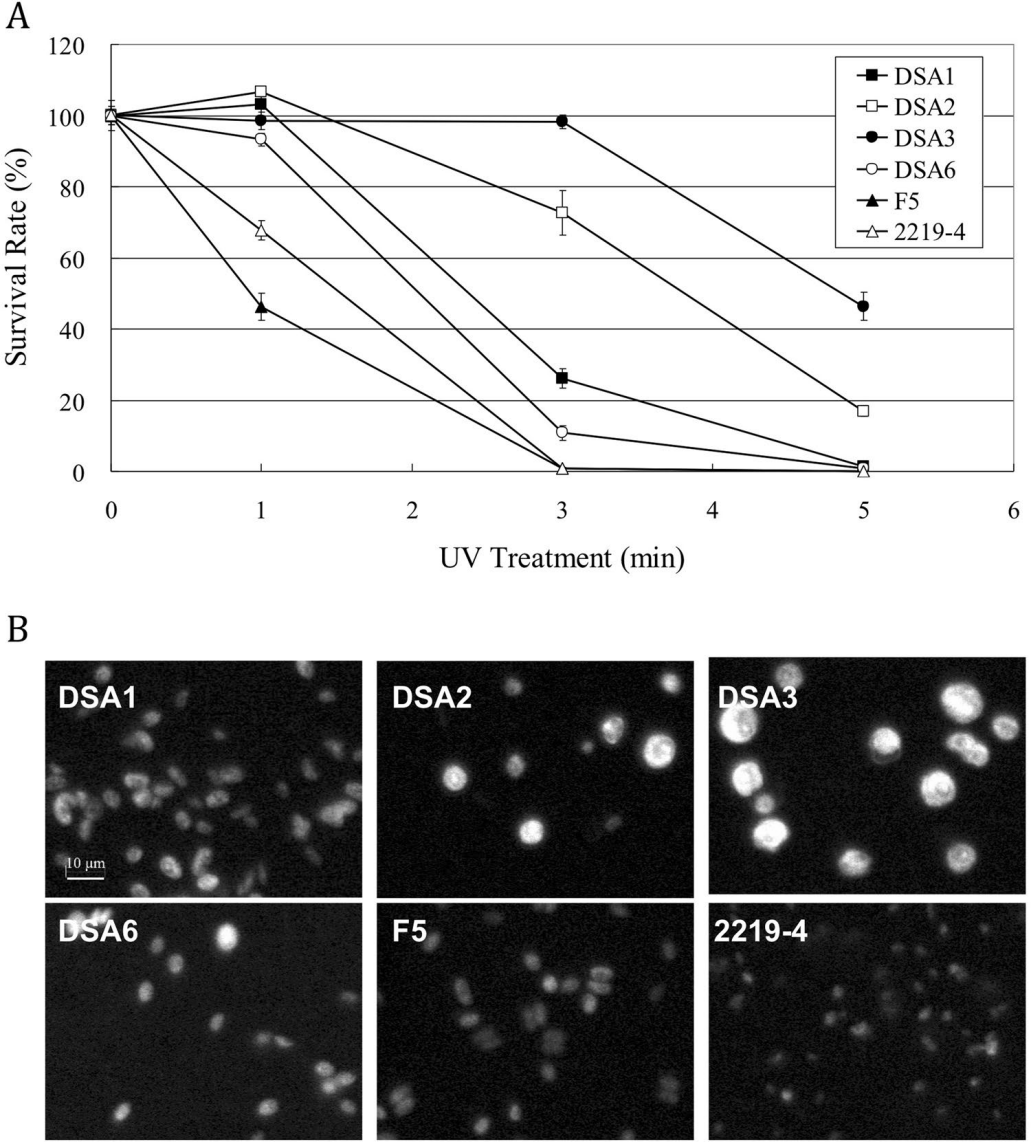
**A** UV-B stress tolerance of the four airborne microalgae and the two waterborne microalgae used as controls. About 500 cells in the early stationary phase were spread onto each agar plate and irradiated with 302 nm UV for the specified durations. The survival rate of each strain was defined as the colony numbers on the UV-treated plates compared to those on the non-treated plates (n = 6, mean ± SE). F5, *Desmodesmus* sp. F5; 2219-4, *Neodesmus* sp. UTEX 2219-4. **B** Different autofluorescence intensities from the cell wall of the six microalgal strains.

When traveling in the air, airborne microalgae are inevitably exposed to much higher levels of UV radiation compared to microalgae in the aquatic environments. In order to survive aerial travel, these cells must have a mechanism(s) to protect themselves against the damaging effects of the radiation, a mechanism that is of less concern to waterborne microalgal populations. To verify whether the four airborne microalgae resisted UV radiation better than waterborne microalgae, these microorganisms were spread onto agar plates using the top agar method^11^, and exposed to UV-B radiation to determine their survival rates. The survival rates were compared to those of the two waterborne microalgae, *Desmodesmus* sp. F5^12^ and *Neodesmus* sp. UTEX 2219-4^13^ (hereafter referred to as F5 and 2219-4, respectively), both are members of Scenedesmaceae as well. As shown in Fig. 2A, the survival rates of the two waterborne microalgae were about 68 and 46%, respectively, after 1 min of the UV-B exposure, compared to more than 90% for all four airborne microalgae. After 3 min of the exposure, however, all of the waterborne cells died but the survival rates of the airborne cells were about 98, 73, 26, and 11% for DSA3, DSA2, DSA1 and DSA6, respectively. Significant differences could still be observed among the survival rates of DSA3, DSA2, and the other four species after 5 min of the UV-B exposure.

For algal cells, cell wall is the first barrier to defend the cells against UV attack. It is well known that the cell walls of terrestrial plants are autofluorescent when excited by UV^14^. In this context, the four airborne microalgal strains would have better UV tolerance if their cell walls could absorb UV, and therefore are autofluorescent as well. To examine this possibility, the pigment-free cells were examined under an epifluorescence microscope. Indeed, as shown in Fig. 2B, the cell walls of the four airborne and the two waterborne green microalgae were able to emit autofluorescence when excited by UV under the microscope. Furthermore, the autofluorescence intensities emitted from the six microalgal strains varied, with DSA3 and DSA2 being the strongest, DSA1 and DSA6 in the middle, and F5 and 2219-4 the weakest as detected using the same parameters. The varied intensities suggested that these microalgae had different levels of UV tolerance because their cell walls absorbed different amounts of UV energy.

### The UV tolerance of the microalgae was positively correlated with their cell wall thickness

**Figure 3:**
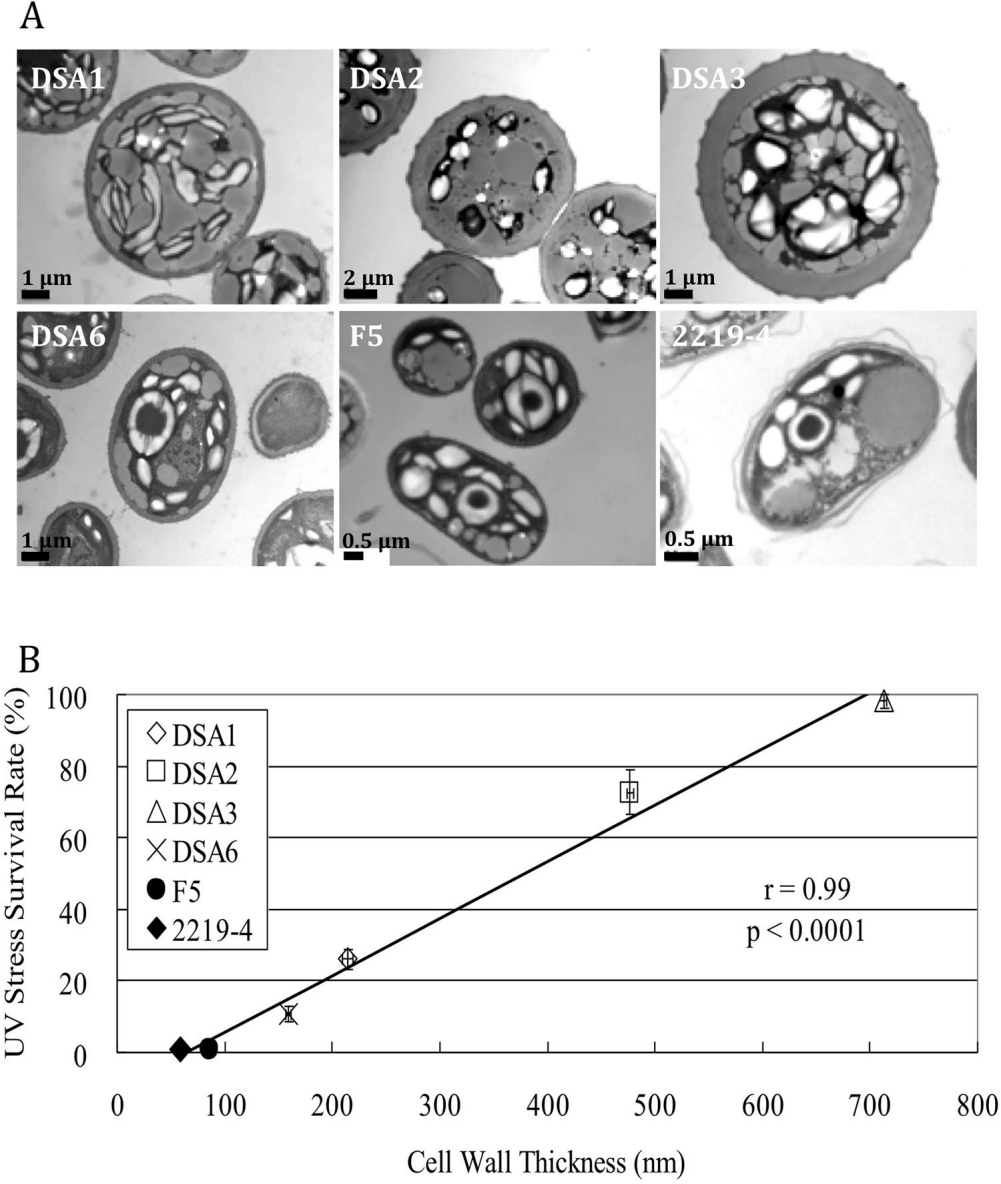
**A** Transmission electron microscopy (TEM) micrographs of the four airborne microalgae and the two waterborne microalgae used as controls. These cells were sampled in the early stationary phase. Note the differences in the cell wall thickness among these strains. **B** The correlation between the UV-B stress survival rates and the cell wall thickness of the six microalgal strains (r = 0.99, *p* < 0.0001, analyzed using the StatView Correlation Coefficient method). The cell wall thickness of each strain was measured based on the TEM images (n = 10, mean ± SE).

There appeared to be a correlation between the autofluorescence intensities emitted by the cell walls and the survival rates of the six microalgal strains exposed to the UV-B radiation for 3 min. It was intriguing to investigate whether the cell wall thickness of these microalgae played a role in the survival rates. To measure the cell wall thickness of the six microalgal strains, these cells were fixed and sectioned, and then observed using Transmission Electron Microscopy (TEM). As shown in Fig. 3A, the cell wall thickness of the six strains varied, with DSA3 and DSA2 being the thickest, DSA1 and DSA6 intermediate, and F5 and 2219-4 the thinnest. This relation well-correlated with that of the autofluorescence intensities from their cell walls. When the survival rates under 3 min of the UV-B exposure were plotted against the cell wall thickness of the six strains, a good correlation (r = 0.99, *p* < 0.0001, Correlation Coefficient method in StatView) was revealed (Fig. 3B). This finding suggests that a thicker cell wall, which absorbs more UV energy and emits stronger autofluorescence, provides better protection against UV radiation in the case of green microalgae.

### The four airborne green microalgae survived the freeze–desiccation stress treatment

**Figure 4:**
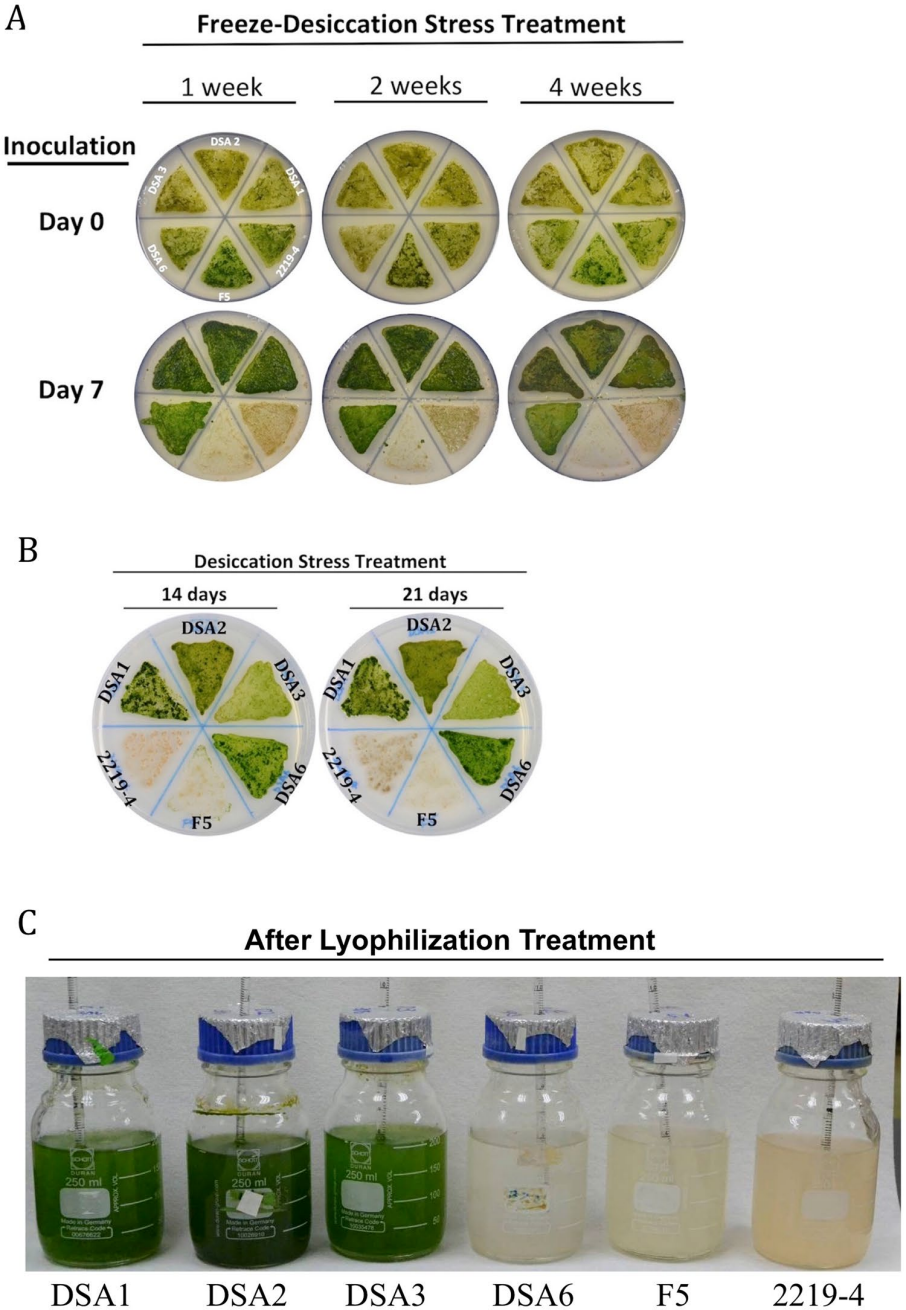
**A** Vitalities of the six microalgal strains after freeze–desiccation stress treatments. Early stationary phase cells of the six microalgal species were air-dried on the bench and placed at − 20 °C for 1, 2 and 4 weeks before spread onto the agar plates. All the four airborne microalgae DSA1, DSA2, DSA3, and DSA6 grew into dense cell lawns in 7 days but the two waterborne species died after these stress treatments. **B** Vitalities of the six microalgal strains after desiccation stress treatments. Early stationary phase cells of the six strains were air-dried on the bench and placed at room temperature in the dark for 14 or 21 days before spread onto the agar plates. The four airborne microalgae DSA1, DSA2, DSA3, and DSA6 grew into dense cell lawns in 7 days but the two waterborne species died after the stress treatments. **C** Vitalities of the six microalgal strains after lyophilization treatment. Early stationary phase cells of the six microalgal strains were air-dried on the bench and lyophilized for 16 h. One hundred mg of the treated cells of each strain was inoculated into a bottle and allowed to grow for 7 days. The three airborne microalgae DSA1, DSA2, DSA3 grew into dense cultures but the airborne species DSA6 and the two waterborne species died after the stress treatments.

In addition to UV radiation, airborne microalgae are also subjected to desiccation and extreme low temperature stresses especially dispersed in winter. To examine whether the four airborne strains had better tolerance to the two stresses than the waterborne microalgae, the six strains were grown in the B3N liquid medium until early stationary phase. These cells were collected, transferred onto nylon membranes and air-dried on the bench. The samples were then placed at − 20 °C in the dark for 1, 2 and 4 weeks and at the end spread onto agar plates to examine their vitalities. As shown in Fig. 4A, the cell lawns of the four airborne microalgae grew denser in 7 days in the incubation compared to those on day 0. On the other hand, the cells of the two waterborne species died after the double stress treatment. In the less challenging conditions, the air-dried cells of the six strains were placed at room temperature in the dark for 14 and 21 days, and spread onto agar plates (Fig. 4B). Again, the two waterborne species could not survive the desiccation stress while the four airborne strains grew well.

The strong tolerance of the four airborne microalgae prompted the question whether these cells could survive the harsh conditions in the lyophilization chamber that had extreme low temperature, air pressure and moisture that were harsher than the conditions they could encounter during traveling at high altitudes in the air? To answer this question, the air-dried cells of each species were lyophilized for 16 h before inoculated to bottles containing the B3N medium. As shown in Fig. 4C, DSA1, DSA2 and DSA3 grew well in the cultivation but DSA6 and the two waterborne microalgae failed to grow after the treatment.

### The thick cell walls did not confer water retention in the airborne microalgal cells

**Figure 5:**
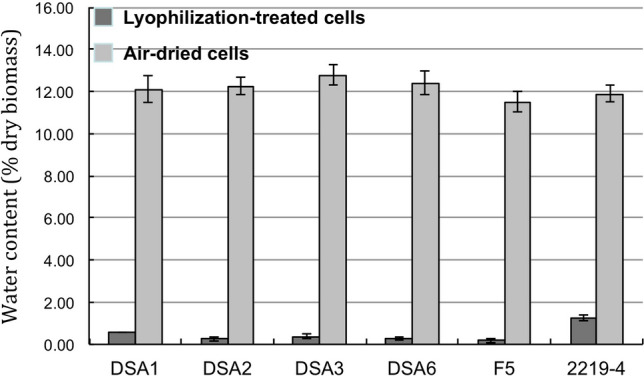
Water content of the lyophilized and the air-dried cells of the six microalgae (n = 3, mean ± SE).

Since the cell wall of airborne DSA3 and DSA2 is much thicker (average 713 and 477 nm, respectively) than that of the two waterborne species (average 85 and 59 nm for F5 and 2219-4, respectively) and their desiccation tolerance is also much better, the question whether the thick cell wall played a role in water retention in the airborne microalgae arose. To determine the water content of these air-dried and the lyophilized cells, the weight of each sample was measured before and after heating at 105 °C for 24 h using a high accuracy analytical balance. As shown in Fig. 5, the water contents of the air-dried cells of the six strains were about 12%, which did not explain their differences in the desiccation tolerance. The water content of the lyophilized cells was below 1% with the exception for 2219-4. This did not explain their survival rates after the lyophilization treatment either.

### Total antioxidation capacities of the airborne microalgae did not confer their freezing and desiccation tolerance

**Figure 6:**
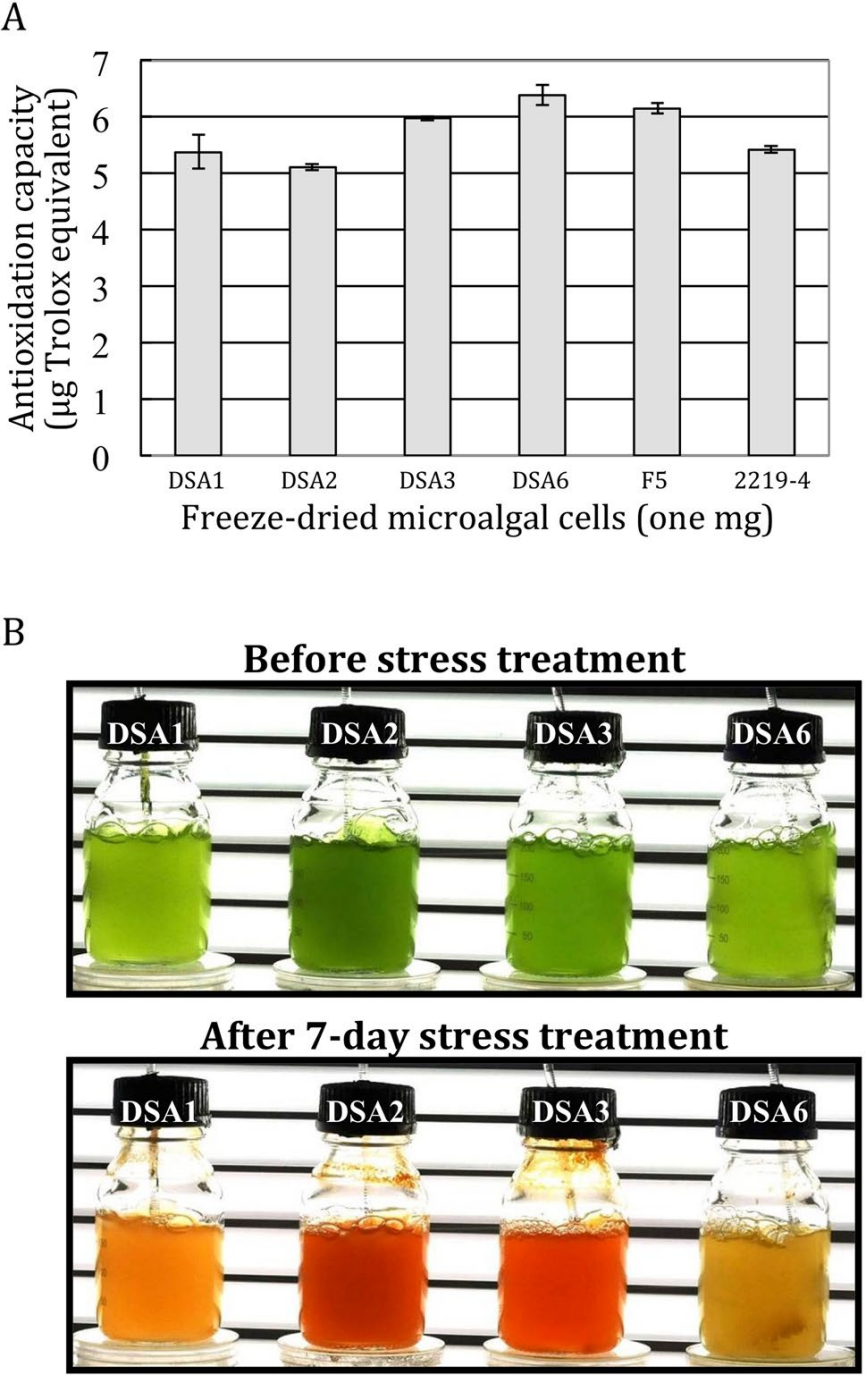
**A** Antioxidation capacity of the six microalgal extracts from the early stationary phase cells. One mg lyophilized microalgal cells of each species was analyzed (n = 3, mean ± SE). Antioxidation capacity is expressed as the Trolox equivalent. **B** Production of carotenoids by the four airborne microalgae before and after the stress treatment. Upper panel, cells in the early stationary phase; lower panel, cells after nitrogen deprivation and 1.5% NaCl stress under high light intensity (800 μmole photo/m^2^/s) treatment for 7 days.

Intracellular oxidative stress is common in photosynthetic cells under environmental stress. Eukaryotic cells in general are equipped with various mechanisms to ameliorate oxidative stress. One mechanism to prevent damage caused by reactive oxygen species (ROS) such as superoxide, hydrogen peroxide and hydroxyl radicals in photosynthetic cells is production of antioxidants in the cells to detoxify these harmful molecules. To examine whether the levels of antioxidants in the airborne microalgae contributed to their better freezing and desiccation tolerance, the total antioxidation capacities of the six strains in the early stationary phase were compared. As shown in Fig. 6A, the total antioxidation capacity of DSA3, the strain with strong tolerance to the stresses tested, was at the same level as that of F5, the waterborne species with weak tolerance. The antioxidant levels of the other strains did not show dramatic difference either. Therefore, their differences in freezing and desiccation tolerance demonstrated in this study did not appear to be conferred by their total antioxidation capacities.

### The airborne microalgae produced carotenoids under prolonged stressful conditions

Colonies of the airborne microalgae became reddish on agar plates after prolonged incubation (more than 4 weeks) under continuous light. This phenomenon is reminiscent of *Haematococcus pluvialis*^15^, *Coelastrella striolata*^16^ and *Coelastrella* sp. F50^17^, which accumulate high levels of carotenoids, which are strong antioxidants, under stressful conditions. Two of the four airborne microalgae are members of *Coelastrella*, and they form a clade with *Coelastrella* sp. F50 (Fig. 1), suggesting they are able to produce carotenoids as well. To verify this view, the four airborne microalgae were cultivated in the liquid medium under normal conditions until reaching stationary phase. These cells were then subjected to nitrogen starvation and salt stress under high light intensity. As shown in Fig. 6B, these cells changed color from green to red or yellow–brown in seven days of this stress treatment. HPLC analysis of these pigments of DSA2 and DSA3 demonstrated that the pigments contained carotenoids including astaxanthin, canthaxanthin and β-carotene similar to the pigments identified in *Coelastrella* sp. F50^17^.

## Discussion

It is now well known that fine particles can be transported across long distances, such as cross-ocean or continent, by strong wind. Darwin’s report was one good example. It is also well known that viable microalgae and cyanobacteria exist in the air, even in the high altitudes that can be collected using an airplane. This study brought our understanding of microalgal dispersal one step further, which is some species have evolved to gain the potentials to survive the stresses encountered in the long distance aerial dispersal in the wind.

It is interesting to note that *Coelastrella* spp. DSA2 and DSA3, whether the same species to be determined, form a clade with *Coelastrella* sp. F50, a species previously isolated from southern Taiwan^17^. This clade differs from the European *Coelastrella* clade that comprises *C. striolala*, *C. corcontica*, *C. aeroterrestrica*, *C. terrestris* CCALA 476, and *C. terrestris* CAUP H 4403^18^ based on the analysis of ITS1-ITS2 fusion sequences (Fig. 1B). This observation suggests that *Coelastrella* spp. F50, DSA2, and DSA3 share a common evolutionary history in East Asia, which is parallel to that of the European *Coelastrella* spp.. The two Dongsha Atoll isolates are reminiscent of the aerial *Coelastrella* spp*.* and *Scenedesmus* spp*.* isolated by Abe et al*.* from rock surfaces in Japan (their ITS sequences not available at the time of this manuscript preparation), which could produce high levels of carotenoids^16,19,20^. The common features of DSA2, DSA3, and the two aeroterrestrial *Coelastrella* spp*.* isolated by Abe et al*.* coupled with their divergence suggest that the genus *Coelastrella* has adapted to hard substrate environments for a long period of time. Their work also confirmed that *Coelastrella* spp. are able to cope with UV radiation and desiccation stresses that are common on rock surfaces. The tolerance to these stresses potentiates *Coelastrella* spp. to become airborne microalgae.

In addition to being airborne, one possibility for freshwater microalgae to arrive at this tiny coral sand island is to be transported by migratory birds^21^. However, due to the lack of freshwater pond and river, the chance for freshwater birds to stop over on this island is rare. Freshwater birds can transport microalgae with or without traits of UV, desiccation and freezing temperature tolerance, since these birds can provide protection to their passengers. Therefore, it is possible but unlikely for all the four Dongsha strains, if they were transported by freshwater birds, to have these traits and none of them is similar to waterborne microalgae. Thus, to arrive at this island, at least part of the four strains must be brought by the wind if not all.

The stress conditions used in this study, such as the UV intensity, desiccation and low temperature stresses, were harsher than those airborne microalgae could encounter in the natural environment. Continuous illumination, which is a mild stress to photosynthetic cells, was also used instead of 12/12 h light/dark cycling for the microalgal cultivation. The purpose of these stress treatments was to challenge the stress tolerance of these airborne and waterborne microalgae, and obviously these treatments differentiated these microalgae well. UV-B is more destructive to cells than UV-A due to its higher energy. UV-B is absorbed by the ring structures of DNA and protein molecules. It damages cells by creating lesions in the DNA sequences and producing reactive oxygen species (ROS). The cells would be killed if the lesions are widespread or the reactive oxygen species are not quickly detoxified. The possible mechanisms to resist UV include compounds that can absorb UV before it causes irreversible damages, ROS scavenging mechanisms including enzymes and small antioxidative molecules, and rapid DNA repair in the nuclei^22^. These airborne microalgal strains presented in this work had multiple layers of protection against UV stress, including thick cell walls, carotenoids, and other antioxidant molecules. On the other hand, the thick cell walls do not appear to have an effect on retaining water in the cells, nor does the airborne microalgae keep water in the cells by accumulating high levels of sugars as osmolytes as in some land plants, since the water contents in the air-dried cells of the six strains were about the same. This suggests that these airborne species have other mechanisms to protect their protein and biomembrane when the cells are desiccated.

Both freezing and desiccation stresses are able to cause structural damages and physiological arrest in the cells^23^. For example, under these stresses, the arrangement of phospholipids in the biomembrane is disturbed and therefore membrane leakage occurs. Proteins such as enzymes, receptors, transporters, etc., undergo conformational changes and thus their functions are impaired or lost. In this situation, metabolism is slowed or stopped, and ATP biosynthesis can cease. Intracellular oxidative stress is common when photosynthetic cells are under environmental stresses, and ROS can severely damage the cells if they are not quickly detoxified^24–26^. All these problems can cause cell death if they are not resolved in time. Studies on the tolerance against desiccation and freezing stresses in land plants have made significant progress. Plants with desiccation tolerance are often resistant to freezing stress^27,28^, a phenomenon called cross-tolerance. Therefore, the evolution of desiccation tolerance and freezing tolerance in these airborne green microalgae might not be independent events. Transcriptome sequencing will give much information about how the cellular protections are provided. Knowledge of these mechanisms might be useful for crop breeding considering global climate change that results in frequent drought conditions in many regions in the world^28^.

Before airborne microalgae can be blown into the atmosphere by strong wind from their habitats such as tree barks, rooftops, building walls, rock surfaces or topsoil, there must be a slow cell dehydration process, in the sun light or not, to reduce the cell weight. This process would induce the biosynthesis of carotenoids that serve as a protection agent. Our results showed that the four strains of airborne microalgae could survive the freeze–desiccation stress for at least 4 weeks (Fig. 4A). The speed of the winter monsoon blowing into Dongsha Island is mostly between 10 and 30 km/h. The time required for the monsoon to bring particles from Hokkaido, Japan, which is about 3,500 km away in the north, to Dongsha Island would be between 5 and 14 days. Therefore the stress tolerance of the four strains is strong enough to allow the dispersal from Northeast Asia to Southeast Asia in the winter monsoon.

The physiological roles of carotenoids have been studied intensively. One of the roles includes protection of cells against oxidative stress when they are under environmental stress^29^. Desiccation stress is accompanied by ROS production in the cells, which can severely damage them. Whether the ROS detoxification enzymes such as superoxide dismutase (SOD), glutathione peroxidase (GPX), and ascorbate peroxidase (APX) can work in dehydrated cells is still a question, microalgae do contain an array of small antioxidative molecules including phenolic compounds, flavonoids, and carotenoids to combat ROS^30,31^. It appears that, for the four strains of airborne microalgae to be able to travel a long distance in the air, they had been equipped with thick cell walls and abundant carotenoids before being blown into the air. The cell wall absorbed the UV and prevented the damage it might cause during the journey, the carotenoids detoxified the ROS that might occur under freeze–desiccation conditions and shielded from the high light and UV damages at the high altitudes. It is worth noting that carotenoids are actually dual-functional. They effectively absorb blue light and UV to a lesser degree, and function as antioxidants. When the cellular conditions are not suitable for photosynthesis and the light intensity in the environment is high, carotenoids can effectively absorb blue light, the high energy for photosynthesis, and dissipate the energy as heat to protect the photosystems.

How do airborne microalgal cells prevent biomembrane disruption and protein denaturation caused by the stresses in the long distance dispersal is still not clear. It is unlikely that thick cell wall and carotenoid accumulation were able to provide these protections. More studies are necessary to investigate other mechanisms that are used by airborne microalgae to cope with desiccation and freezing stresses. The approaches would include mutagenesis and looking for non-tolerant mutants followed by comparative proteomics study. It would be also interesting to study whether red algae and brown algae have similar mechanisms encoded in their genomes. The comparative study would help explain how green algae could adapt to the dry land about 450 million years ago but not red and brown algae.

## Materials and methods

### Isolation and cultivation of airborne microalgae

Agar plates (1.5%) prepared with 0.2 g/L Hyponex fertilizer (30-10-10) (Marysville, OH, USA) using freshwater were attached to a stainless steel tray (50 × 30 cm) using Velcro tapes. The tray was fixed on a tall rack (Supplementary Figure 2), which was inserted into the northern beach of the Dongsha Island (20 °43′ N, 116° 42′ E). The tray was two meters above ground, and the open agar plates faced north to receive the northeastern monsoon in December 2013. The agar plates on the tray were replaced every day, and the collection lasted for a month. The inoculated plates were sealed with the lids and incubated under continuous 80 μmol photon/m^2^/s at 28 °C to grow colonies. Single colonies of green microalgae were picked up and the cells were first grown in one mL of a modified Bold 3 N (B3N) freshwater medium containing 4.4 mM NaNO_3_, 0.17 mM CaCl_2_, 0.3 mM MgSO_4_, 0.22 mM K_2_HPO_4_, 0.65 mM KH_2_PO_4_, 0.43 mM NaCl, and the minerals described in Berges et al.^32^ in culture tubes. The culture volume was serially scaled up to 10, 100 and 1,000 mL in PYREX bottles later. Plentiful air filtered through a membrane (pore size 0.2 μm, Sarstedt, Germany) was pumped into the cultivation bottles, and the cultures were illuminated continuously with 150 μmol photon/m^2^/s from one side of the bottles. These isolated microalgal strains grown in the bottles were also maintained on 1.5% agar plates prepared with the modified B3N medium under continuous 80 μmol photon/m^2^/s at 28 °C. Colonies on these plates were also used for long-term observation of phenotype and pigment production.

### Electron microscopy

Scanning electron microscopy (SEM) was carried out using the procedures described in Tsai et al.^33^ with modifications. Early-stationary phase cells were fixed with 2.5% glutaraldehyde at room temperature for four hours. After removing the fixative, the cells were rinsed with reverse-osmosed water three times. The re-suspended cells were dropped onto a small piece of 0.45 μm nylon membrane (0.5 × 0.5 cm). When the cells settled on the membrane, the sample was dehydrated in serial 30, 50, 70, 85, 95 and 100% ethanol, 10 min at each step, and finalized with 100% acetone. The procedures for the critical point drying, coating and scanning were the same as those described in Tsai et al. The procedures for transmission electron microscopy (TEM) were described in Hu et al.^17^. The cell wall thickness was measured using the scale bars on each photo.

### DNA extraction, PCR, sequencing and phylogenetic analysis

These procedures of DNA extraction, PCR, sequencing and phylogenetic analysis were described previously^33,34^. The primers for ITS1-5.8S-ITS2 rDNA fragment PCR amplification were forward 5′-ACCTAGAGGAAGGAGAAGTCGTAA-3′ and reverse 5′-TTCCTCCGCTTATTGATATGC-3′. The primers for 18S rDNA PCR amplification were forward 5′-TGATGGTACCTACTACTCGGA-3′ and reverse 5′-ACGGGCGGTGTGTACAAA-3′. DNA sequencing was conducted using the dideoxy method with the primers above. Phylogenetic analysis was carried out using the Maximum Likelihood method of the Mega 5 software^35^. The matrix of the fused ITS1-ITS2 sequences comprised 286 positions.

### Preparation of pigment-free cells and imaging of cell wall autofluorescence

Microalgal cells in the stationary phase were frozen at − 20 °C and thawed for three cycles. Pigments were extracted by using 95% ethanol with 1% Triton at 75 °C repeatedly until the cells became white. The extraction was finalized with the same solution at room temperature with gentle mixing overnight. The cell wall autofluorescence of the microalgal samples was imaged with an epifluorescent microscope (Axio Scope A1, Zeiss, Germany) using the DAPI filter and the AxioCam ICc1 digital camera. Samples with weak autofluorescence intensities were imaged first to make sure the signals were visible. Other samples were imaged successively using the same parameters set up in the software for the imaging work.

### UV stress treatment and survival rate measurement

The microalgal cells collected from liquid cultures were spread onto agar plates using the top agar method followed by the UV stress treatment^11^. Briefly, the cells in the early stationary phase, adjusted to approximately 500 cells/mL counted using a particle size analyzer (Beckman-Coulter Company, Indianapolis, IN, USA), were suspended in 0.2% agar. One mL of the suspended cells was transferred to an agar plate and spread to cover the whole plate. These agar plates covered with cells were irradiated with UV-B (302 nm, 6.5 mW/cm^2^) using a UV box (LM-20E Benchtop UV Transilluminator, UVP Company, Upland, CA, USA) for 1, 3 or 5 min, and then incubated under continuous 80 μmol photon/m^2^/s at 28 °C to until the colonies were clearly visible. The colonies were counted with the “analyze particles” function of the Image J software (Version 1.49 V). The survival rates of the UV stressed cells on the plates were estimated with the controls without UV treatment. The top agar method for spreading single cells was not suitable for the air-dried and the lyophilized cells because they formed small aggregates.

### Treatments of freezing and desiccation stress and vitality tests

To execute the freeze–desiccation stress test, cells in the early stationary phase were collected using slow centrifugation (2000×*g*, 10 min at room temperature), transferred onto a nylon membrane with paper towels beneath for air-drying on the bench under dim light for 24 h. The air-dried cells were then placed at − 20 °C in the dark for the specified durations indicated in the results. Five mg of the cells after the freeze–desiccation treatment were re-suspended in 20 μL of the B3N medium and spread onto agar plates prepared with the B3N medium. The plates were incubated under 80 μmol photon/m^2^/s at 28 °C for 7 days to examine the vitalities of the cells. To execute the desiccation stress test, the cells were collected and air-dried as described above, and then placed in the dark at room temperature for the durations specified in the results. The cells were then spread onto the agar plates to examine their vitalities as described above. To execute the lyophilization stress test, the cells were collected and air-dried as described above and placed in the lyophilization chamber (200 mTorr, − 50 °C, near-zero moisture) for 16 h. One hundred mg of the lyophilized cells of each species were inoculated into one bottle containing the B3N medium. The cells were cultivated for 7 days in the conditions described above to examine their vitalities. Biomass of the air-dried and the lyophilized cells was weighed using a high accuracy analytical balance (METTLER AT21, Columbus, OH, USA; readability to 5 μg).

### Antioxidation capacity assay

The Trolox equivalent antioxidant capacity (TEAC) assay was used in this study with some modifications^31,36^. Briefly, lyophilized microalgal samples were extracted with 75% ethanol using a mini-beadbeater (BioSpec Products, USA) at the highest speed for 5 cycles, 20 s each, and cooled down on ice for 2 min between two cycles. Under dim light, ABTS (A-1888, Sigma-Aldrich, USA) and potassium persulfate (216,224, Sigma-Aldrich, USA) were dissolved in water to generate the blue-green ABTS^·+^ (7 mM ABTS and 2.45 mM potassium persulfate). This mix was allowed to react in the dark at room temperature for 16 h and its absorbance at 734 nm (OD_734_) was adjusted to 0.70 ± 0.05 before use. To build the standard curve of OD_734_ against Trolox quantity, different amounts of Trolox (238813, Sigma-Aldrich, USA) were mixed with the freshly prepared ABTS^·+^ solution to reduce the color, and the resulting OD_734_ readings were recorded. The antioxidation capacity of the samples was expressed as equivalent amount of Trolox per mg dry biomass.

## Supplementary information

Supplementary Legends.

Supplementary Figures.
