# Supplementary material for: Mechanisms protect airborne green microalgae during long distance dispersal
Source: Sci Rep. 2020 Aug 19;10:13984. doi: 10.1038/s41598-020-71004-y (PMC7438330; doi:10.1038/s41598-020-71004-y)
Supplement: Supplementary file 1 — Supplementary Legends. [file 41598_2020_71004_MOESM1_ESM.docx]

**Supplementary Figure 1**. A typical wind map in East Asia in the winter (Source: Windy.com. Use of this map was granted under the “Permission to Use Visualizations, General Terms of Use, Paragraph 9”). Wind directions in other seasons are more variable due to the changing locations of the atmospheric high-pressure systems.

**Supplementary Figure 2**. The airborne microalgae trap used in this study. Agar plates prepared with a freshwater medium were fixed to the stainless steel tray using Velcro tapes.
