# Supplementary material for: Mechanisms protect airborne green microalgae during long distance dispersal
Source: Sci Rep. 2020 Aug 19;10:13984. doi: 10.1038/s41598-020-71004-y (PMC7438330; doi:10.1038/s41598-020-71004-y)

Mechanisms protect airborne green microalgae during long distance dispersal

Authors
Chia-Sheng Chiu[1], Pai-Ho Chiu[1], Tze Ching Yong[1], Hsin-Pei Tsai[1], Keryea Soong[1], Hsiang-En Huang[2] and Ching-Nen Nathan Chen[1*]

*Corresponding author
Email: nathanc@mail.nsysu.edu.tw

Affiliations
[1]Department of Oceanography, National Sun Yat-sen University, Kaohsiung, 804, Taiwan

[2]Department of Life Science, National Taitung University, Taitung, 950, Taiwan

Supplementary Figure 1 Chiu *et al.*
(This is a color figure.)

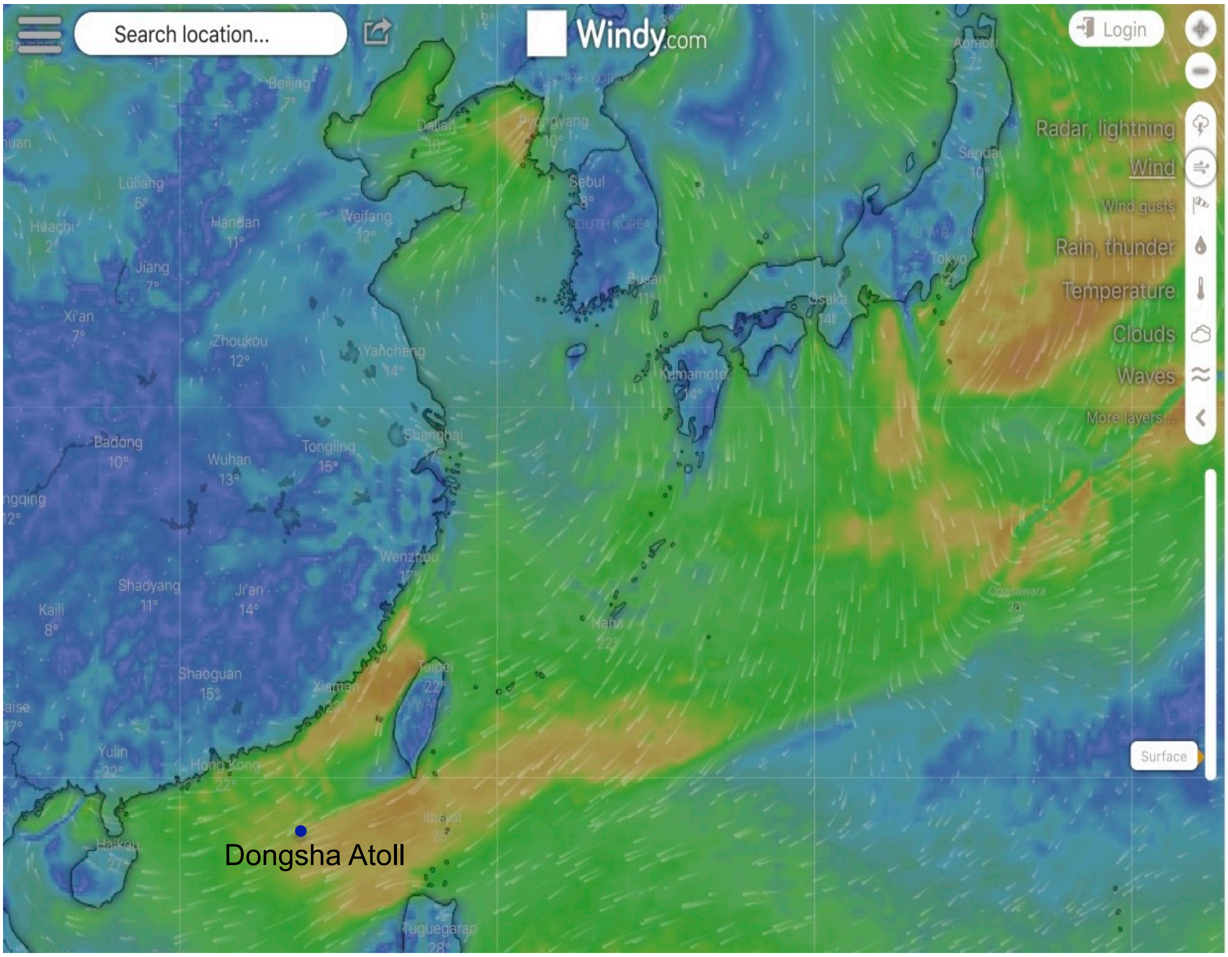

Supplementary Figure 2 Chiu *et al.*
(This is a color figure.)

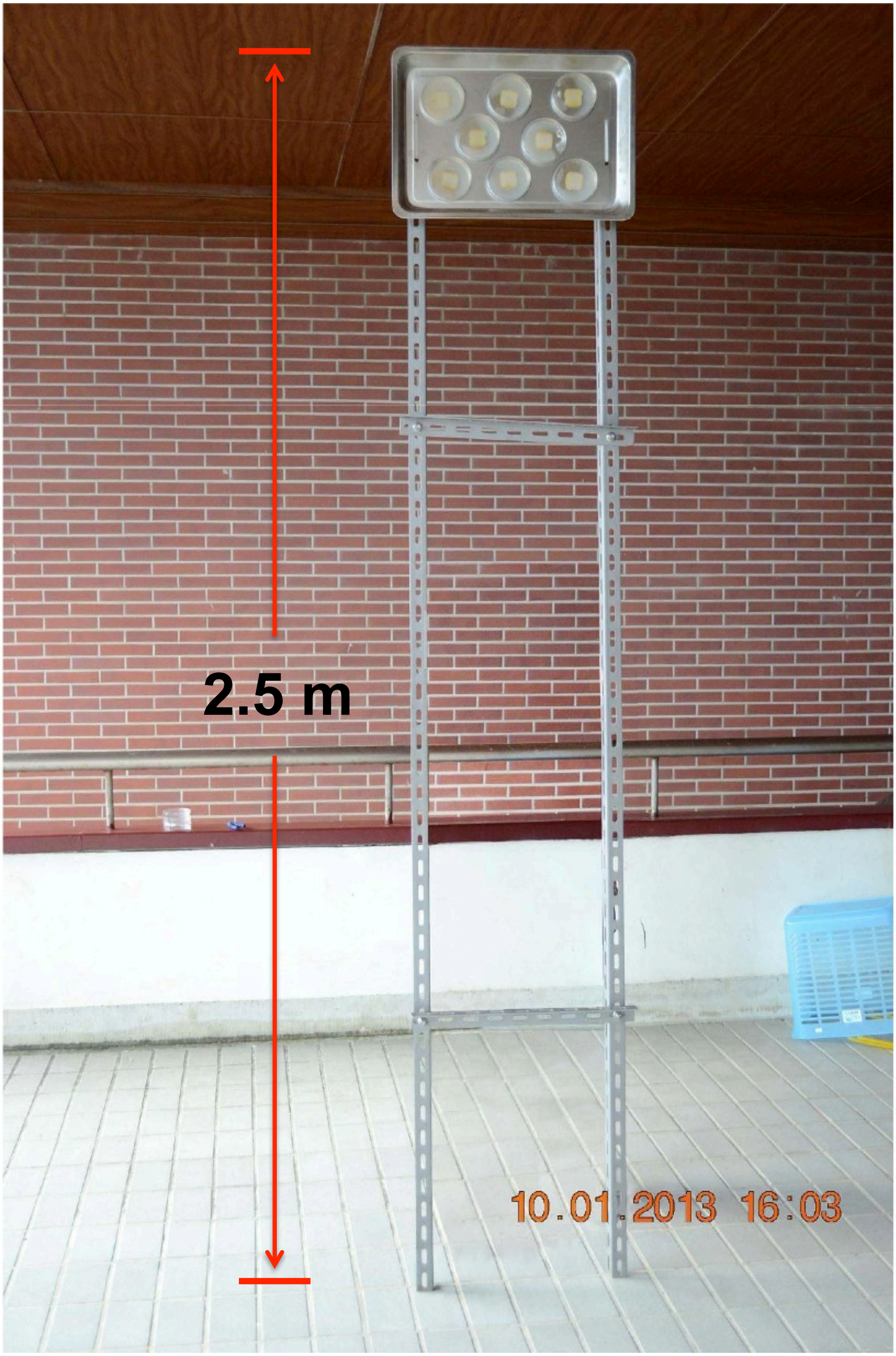

Supplement: Supplementary file 2 — Supplementary Figures. [file 41598_2020_71004_MOESM2_ESM.pdf]
